# Ameliorative effect of selenium yeast supplementation on the physio-pathological impacts ofchronic exposure to glyphosate and or malathion in *Oreochromis niloticus*

**DOI:** 10.1186/s12917-022-03261-0

**Published:** 2022-05-01

**Authors:** Marwa A. Hassan, Samaa T. Hozien, Mona M. Abdel Wahab, Ahmed M. Hassan

**Affiliations:** 1 Faculty of Veterinary Medicine, Department of Animal Hygiene, Zoonoses and Behaviour, Suez Canal University, Ismailia, 41522 Egypt; 2 Animal Health Research Institute, Ismailia, 41522 Egypt

**Keywords:** Selenium yeast, Glyphosate, Malathion - *Oreochromis niloticus*, Performance, Hematology, Oxidative stress

## Abstract

**Background:**

Pesticide exposure is thought to be a major contributor to living organism health deterioration, as evidenced by its impact on both cultured fish species and human health. Commercial fish diets are typically deficient in selenium (Se); hence, supplementation may be necessary to meet requirements during stress. Therefore, this study was conducted to investigate the protective role of selenium yeast (SY) supplementation for 60 days against the deleterious effects of glyphosate and or malathion chronic toxicity at sublethal concentrations in *Oreochromis niloticus* .

**Methods:**

Two hundred and ten fish were divided into seven groups (*n* = 30/group) as follows: G1 (negative control); G2 (2 mg L^− 1^ glyphosate); G3 (0.5 mg L^− 1^ malathion); G4 (glyphosate 1.6 mg L^− 1^ and malathion 0.3 mg L^− 1^); G5 (glyphosate 2 mg L^− 1^ and SY 3.3 mg kg^− 1^); G6 (malathion 0.5 mg L^− 1^ and SY 3.3 mg kg^− 1^); and G7 (glyphosate 1.6 mg L^− 1^; malathion 0.3 mg L^− 1^ and SY 3.3 mg kg^− 1^).

**Results:**

Results revealed significant alteration in growth performance parameters including feed intake (FI), body weight (BW), body weight gain (BWG), specific growth rate (SGR), feed conversion ratio (FCR), and protein efficiency ratio (PER). G4 has the highest documented cumulative mortalities (40%), followed by G3 (30%). Additionally, the greatest impact was documented in G4, followed by G3 and then G2 as severe anemia with significant thrombocytopenia; leukocytosis; hypoproteinemia; increased Alanine aminotransferase (ALT) and Aspartate aminotransferase (AST), urea, and creatinine, as well as malondialdehyde (MDA), superoxide dismutase (SOD) and glutathione peroxidase (GPx). Considering the previously mentioned parameters, selenium yeast (*Saccharomyces cerevisiae*) (3.3 mg kg^− 1^ available selenium) mitigated the negative impact of both the agrochemicals, whether exposed singly or in combination, in addition to their antioxidative action.

**Conclusions:**

In conclusion, our study found that organophosphorus agrochemicals, single or combined, had negative impacts on *Oreochromis niloticus* regarding growth performance, biochemical and hematological changes in the serum, as well as induced oxidative damage in liver and kidney tissues. Supplementation of SY at the rate of 3.3 mg kg^− 1^ diet (2.36 mg kg^− 1^ selenomethionine and 0.94 mg organic selenium) ameliorated the fish performance and health status adversely affected by organophosphorus agrochemical intoxication.

## Background

Egyptian aquaculture production has increased significantly in the last decade, and Egypt is currently the leading producer in Africa. Egypt is the world’s ninth-largest aquaculture supplier, producing over 1.6 million tonnes in 2019, with Nile tilapia accounting for the overwhelming majority (66%) [1]. The most farmed fish is Nile tilapia, and Egypt is the world’s second-largest producer of farmed tilapia after China, with a total value of over 900,000 USD [2]. Grey mullet and carp are frequently farmed, often in combination with tilapia, and together these species represent more than 95% of Egyptian aquaculture production [2]. Egypt is one of the countries with restricted water resources with limited volume and quality of water available for fish farming [3]. The aquaculture business, regardless of size, is not permitted to utilize irrigation or Nile water and must alternatively depend on water from agricultural drainage canals and groundwater [4]. The Nile Delta area is surrounded by semi-intensive fish culture using both brackish and freshwater, which is Egypt’s most significant farming technology accounting for 86% of aquaculture production [2]. The agricultural drainage water polluted with various agricultural chemicals including organophosphates is negatively influencing the quality of farmed fish [5].

Pesticide and herbicide pollutants, particularly run-off from agricultural areas, are a major global concern because of the acute and chronic toxicity to aquatic organisms [6]. Since the ban on organochlorines (OC) due to their continuing harmful effects, organophosphates (OP) have been chosen as the most preferred insecticides in today’s world to make pest-free crops more productive [7]. OP are considered global environmental hazardous substances due to their sustained use. Significant levels of total organophosphorus pesticide residues were detected in aquaculture water (73.57 ± 62.97 ppb), sediment (103.03 ± 16.05 ppb), and fish muscle samples (*Claris gariepinus* 49.1 ± 17.8 ppb, *Tilapia zilli* 48.3 ± 18.9 ppb and *Oreochromis niloticus* (45.6 ± 28.7 ppb) [8]. On March 20, 2015, the International Agency for Research on Cancer (IARC) of the World Health Organization (WHO) categorized two organophosphate insecticides (malathion and diazinon) and one herbicide (glyphosate) as “probably carcinogenic to humans” (Group 2A). However, these two pesticides and glyphosate are widely used in Egypt [9]. Glyphosate traces have been identified in surface waterways in various sites (8.7 ug L ^− 1^ [10], 86 ug L ^− 1^ [11], and 430 ug L ^− 1^ [12], malathion has been estimated by Derbalah and Shaheen [13] in water samples at different fish farms sites and found their concentration ranged from 0.37 to 4.12 μg L^− 1^. Exposure to pesticides, either chronic or acute, could have deleterious impacts on fish performance, physiology, biochemistry, population stability, and the entire ecosystem [14, 15]. Consequently, fish consumption can be identified as a key component of human exposure to these pollutants, indicating their potential risk to human health due to bioaccumulation in farmed fish [16, 17]. Frequent sub-lethal pesticide exposure affects the fish’s growth performance, survival rate, hepato-somatic index, and immunity [18].

N-(phosphoromethyl) glycine, also known as glyphosate, is a weed control herbicide derived from phosphonic acid and glycine that is widely used in agriculture [19]. It is a significant organophosphate (OP), a water-soluble herbicide with a broad spectrum of activity, used to eradicate grass and other unwanted broad-leaf weeds that compete with crops grown worldwide [20]**.** Glyphosate induces hepatic and renal impairment in *Oreochromis niloticus*, and mortality was directly related to exposure dosage; it could be considered highly toxic to Nile tilapias, hence its use near a fish farm or in nearby aquatic environments should be prohibited [21]. Malathion is introduced into the environment at sub-lethal levels, causing serious intimidation as well as severe metabolic disturbances in fish, resulting in a decline and impairment in growth rate and physiological condition [22]. Exposure to malathion at sub-lethal concentrations induced biochemical and hematological alterations in *Oreochromis niloticus* and led to oxidative damage [23].

Selenium (Se) is an important trace element for human and animal species’ successful functionality; however, unlike many other trace elements, it has a limited quantitative range of concentrations between deficiency and physiological conditions and toxic concentrations [24, 25]. A significant amount of research in different animal species has shown the importance of selenium in animals including fish [26] and laying hens for Se-enriched egg production [27]. Selenium can be found in the environment in its inorganic elemental state (Se^0^) as selenides (Se^2−^), selenates (SeO_4_^2−^), or selenites (SeO_3_^2−^) [28], and organic forms as selenomethionine (SeMet) and seleno-cystein (SeCys), [25]. The transformations of selenium depend on various factors such as pH, amount of free oxygen, redox potential, and humidity. Anaerobic conditions, and an acidic environment support the formation of selenium molecules in lower oxidation states, while the higher oxidation states of this element are dominant under aerobic conditions and at alkaline pH [29].

Bioaccumulation of toxic substances triggers redox reactions generating free radicals, especially ROS, which cause physiological alterations in fish tissues [30]. Selenoproteins (SePs) play vital biological roles [31], within cells as components of enzymes including glutathione peroxidase (GPx), deiodinase iodothyronine, and thioredoxin reductase (TRxR), which protect cells from the toxic and harmful effects of free radicals, especially ROS. They also participate in the oxidation of hydrogen peroxide and lipid hydroperoxides as an antioxidant factor [32]. Additionally, Se has been shown to improve performance, counteract reactive oxygen species, and protect the structure and function of proteins, DNA, and chromosomes from oxidative damage [33]. GPx group SePs are predominant in all three domains of life (archaea, bacteria, and eukarya) [34]. The bacteria, protozoa, fungi, and terrestrial plants contain SeCys56-containing GPx sequence homology. GPx carries out a variety of biological roles in cells, including the regulation of hydrogen peroxide (H_2_O_2_), hydroperoxide detoxification, and the maintenance of cellular redox homeostasis [25]. Furthermore, the presence of Se in the active site of GPx affects both its catalytic activity and spatial conformation [23]. Excess selenium can be detrimental to the body; however, reliable measurement of dangerous amounts of selenium is challenging due to the element’s presence in numerous chemical forms [25]. Se toxicity is affected by the Se compound, mode of administration, species of animals, exposure period, idiosyncrasy, physiological condition, and association with other metals, among other factors [35]. Both organic and inorganic forms of selenium can have a detrimental effect on the organism [36]. The dose-dependent selenium toxicity is coupled with competitive inhibition of selenium and Sulphur, resulting in the initiation of Sulphur metabolism (transformation) [37]. Selenium may replace Sulphur in amino acids (cysteine and methionine), while its inorganic form substitutes sulphur during mercapturic acid formation and the interaction of selenites with thiol groups [38]. Therefore, deformed, malfunctioning enzymes and protein molecules could be observed, causing disruptions in the biochemical activity of the cell [39]. High selenium concentrations in the body induce severe hepatic damage, reduced triiodothyronine [T3] levels, and the loss of natural killer cells [40]. Resistance to selenium toxicity is determined by, among other variables, the speed of excretion, and selenium excretion is determined by the rate of methylation of selenium, as discovered in fish [41]. The minimal requirement for Se in livestock is 0.05–0.10 mg/kg dry forage, whereas the toxic Se dosage in animal feed is 2–5 mg/kg dry forage [42]. Selenium methylation detoxifies selenium by forming methyl selenides; nevertheless, an overabundance of selenium in the form of selenocysteine reduces selenium methylation [43]. However, the full decrease of Se to elemental selenium, as accomplished by some bacteria, and the synthesis of heavy metal selenides such as Ag_2_Se or Hg_2_Se result in a non-catalytic, non-toxic form of selenium [44]. Some selenium compounds’ catalytic prooxidant activity appears to be responsible for their toxicity when it exceeds plant and animal methylation processes and antioxidant defenses. Excess selenium can indeed be catabolized into hydrogen selenide and released into the breath, or it can be catabolized into trimethyl-selenium ion and released into the urine [45].

Organic Se supplementation rather than inorganic form had greater absorption, retention rate in fish [46], antioxidant activity, and lower toxicities [47], resulting in less environmental pollution [48]. Besides, Se′s biological function is related to its incorporation into the structure of proteins important for metabolism via SeCys [32]. SeCys can be found in animal tissues and Se-containing proteins, whereas SeMet can be found in yeast, algae, bacteria, and plants [25], and it replaces sulfur in the thiol group (−SH) [24]. Recently, hydroxy-selenomethionine (OH-SeMet) had been synthesized to increase Se bioavailability [49]. The idea behind commercializing Se-Yeast is that it has the potential to supply Se in a more natural dietary form due to its effectiveness and safety [32]. Additionally, it is believed that seleno-compounds in Se-Yeast (SY) are highly bioavailable [50]. Yeast cells can bind organic and inorganic selenium, then bio-accumulates via membrane assembly receptors (extracellularly) and ion transport across the cytoplasmic membrane (intracellularly), then detoxified via oxidation, reduction, methylation, and selenoprotein synthesis processes, allowing yeasts to survive in high selenium concentration culture conditions, implying that selenium yeasts are likely the best absorbers of this element [39]. The use of refined yeast (*S. cerevisiae*) products high in Se is a viable and relatively inexpensive option for Se supplementation [51].

Therefore, the main objective of the study was to evaluate the ameliorative effect of selenium yeast supplementation on the detrimental effects of glyphosate and or malathion on growth performance, hematology, biochemistry, and oxidative stress in *Oreochromis niloticus* after a single or combined chronic exposure.

## Methods

### Chemicals and fish diet preparation

Glyphosate (48% purity) and malathion (57% purity) were purchased from Egypt Kim International Agrochemicals and prepared with distilled water to make a stock solution. The half-life was determined in triplicates (100 L aquarium) at a concentration of 2 mg L^− 1^ glyphosate and 0.5 mg L^− 1^ malathion under the physicochemical parameters of water (22 ± 1 °C and pH 8 ± 0.1) which were to be used for experimental fish exposure to the two chemicals, for chronic toxicity assessment. Samples were collected at 24 h intervals for 72 h and concentrations were assessed by the high-performance liquid chromatography (HPLC) Agilent Series 1200 quaternary gradient pump, Series 1200 autosampler, Series 1200 UV, and fluorescence detector, and HPLC 2D ChemStation software (Hewlett-Packard, Les Ulis, France). The analytical column (stationary phase) was a reversed-phase C18 (250*4.6 mm, 5 μm) Teknorama (Spain). The samples results were processed for probit analysis to calculate the half-life (2.78 and 2.3 days for glyphosate and malathion, respectively) (**data not shown**).

For treatments supplemented with SY, a commercial basal diet was crushed and mixed with 0.8 g selenium yeast (*Saccharomyces cerevisiae*) per Kg of diet (Yeast Sel 2000, Ultra Bio-Logic Inc) containing (2.36 mg kg^− 1^ selenomethionine and 0.94 mg organic selenium). SY was generously provided by Kairouan Group Company, Egypt. The diet was pelletized, spread to dry, and stored at 4 °C for the feeding experiment. Generally, SY-treated and non-treated diets were administered orally to fish at a rate of approximately 3% fish body weight, 2 times /day.

Ingredients (g/Kg^− **1**^ total diet) of basal diet contained: fish meal (300), soybean (350), vitamins and mineral mix (3), corn starch (150), soybean oil (25), wheat bran (25). Chemical composition by proximate analysis (% Dry matter) included: dry matter (94.22), crude protein (42.01), crude lipid (6.3), crude fiber (4.9), ash (7.43), nitrogen-free extract (39.18), and g Adhikari s energy (460 kcal/kg). Vitamin and mineral premix (per kg of mixture) contain the following:15000 IU vitamin A, 1500 IU vitamin D3, 2.0 mg vitamin E, 2 mg vitamin K3, 2.5 vitamin B2, 10 mg vitamin B3, 3 mg vitamin B6, 2 mg vitamin B1, 5 mg vitamin B12, 5.5 mg pantothenic acid, 1 mg niacin, 2 mg folic acid, 100 mg choline, 4 g copper, 300 mg iodine, 30 g iron, 60 g manganese, 50 g zinc, 855.5 g calcium carbonate.

### Water analysis

Some water quality parameters were measured daily [pH, Do and Temperature using Jenway, 370 pH meter, UK and Crison OXI 45 P, EU], twice-weekly [un-ionized ammonia (NH_3_) and Nitrate (NO_3_) following the procedure of spectrophotometric Phenate method and UV screening spectrophotometric method, respectively according to APHA [52] using 1100 Techocomp UV/visible Spectrophotometer], once weekly [Total Hardness (Ethylene diamine tetraacetic acid (EDTA) titrimetric method), Total Alkalinity (titrimetric method), and Chloride (Argentometric Method)]. Sampling procedures and analytical methods for both physical and chemical determinations were carried out according to APHA [52]. Samples were transferred to the laboratory of Hygiene, Zoonoses and Animal Behavior department, Faculty of Veterinary Medicine, Suez Canal University, without delay for immediate measuring.

### Experimental *Oreochromis niloticus*

A total of 250 apparently healthy *Oreochromis niloticus* free from any skin lesions or microbial infections with an average body weight of 14 ± 0.5 g was obtained from nursery ponds at the Central Aquaculture Research Laboratory, Suez Canal University, Ismailia, Egypt. The fish had been acclimatized for 2 weeks in two fiberglass tanks, filled with aerated sterile freshwater with a holding capacity of 1000 *L. prior* to the experiment, the fish were determined to be free of external parasites [53]. The DO was maintained at 5.8 ± 0.02 mg L^− 1^, the water temperature was kept at 22.15 ± 0.17 °C, and a 12 h light/12 h dark**,** photoperiod was adopted [54]. Ammonia (NH_3_) levels in the water were measured 3 times a week recorded as 0.03 ± 0.001 mg L^− 1^. Water quality was optimized meanwhile, periodical water change (30% daily) as per the recommendation of Ahmed, Abdullah, Shuib and Abdul Razak [55] and frequent siphoning of fish wastes were performed. The fish were fed daily to apparent satiety on commercial pellets of 1.5 mL (Skereting 30% protein).

Based on the results of LC_50_-96 h probit analysis (data not shown), the experiment was conducted on a sub-lethal dose by adding LC_15_ (2 mg L^− 1^ for glyphosate and 0.5 mg L^− 1^ for malathion) for single pollutant exposure, and LC_1_ (1.6 mg L^− 1^ for glyphosate+ 0.3 mg L^− 1^ for malathion) for co-exposure of both pollutants, showing alliance with predictions of initial pollution levels found in water samples collected from different fishponds (detected in a previously conducted survey). Briefly, out of the 250 acclimatized fish, 210 apparent healthy fish (14 ± 0.5 g) were randomly assigned into one of seven groups (*n* = 30) with triplicates and exposed to the following treatments: G1 (negative control); G2 (2 mg L^− 1^ glyphosate); G3 (0.5 mg L^− 1^ malathion); G4 (glyphosate 1.6 mg L^− 1^ and malathion 0.3 mg L^− 1^); G5 (glyphosate 2 mg L^− 1^ and SY 3.3 mg kg^− 1^); G6 (malathion 0.5 mg L^− 1^ and SY 3.3 mg kg^− 1^); and G7 (glyphosate 1.6 mg L^− 1^; malathion 0.3 mg L^− 1^ and SY 3.3 mg kg^− 1^)**.** Fish were exposed to the previous treatments for a period of 60 days. In the trial, water was changed every 3 days to simulate field conditions, and pesticide concentrations were adjusted with each water change. Fish were observed daily for any symptoms, and performance parameters were measured and averaged every two weeks starting at 30 days. The following parameters were measured to evaluate both pollutant’s impact and the ameliorative effect of organic selenium.

### Growth parameters and feeding efficiency

Fish anesthetized using clove oil (0.1 mL l ^− 1^) [56] dissolved in ethanol [57] from each aquarium were collected, counted, and bulk weighed periodically (every 2 weeks). Growth performance was determined, and feed utilization was calculated as follows:

Feed intake (FI) was measured biweekly as described [58], body weight (BW), body weight gain (BWG) (g) = final weight – initial weight [59]. Specific growth rate (SGR) was calculated according to the following equations [54]:

$$SGR=\frac{\left(\mathrm{Final}\ \mathrm{weight}-\mathrm{intial}\ \mathrm{weight}\ \right)\times 100}{\mathrm{rearing}\ \mathrm{period}\ \left(\mathrm{days}\right)}$$

Feed conversion ratio (FCR) was estimated according to Fritz et al. (1969) as follows:

$$FCR=\frac{\mathrm{Feed}\ \mathrm{consumed}\ \left(\mathrm{g}\right)}{\mathrm{weight}\ \mathrm{gain}\ \left(\mathrm{g}\right)}\times fish\ number$$

Protein efficiency ratio (PER) was calculated by applying the following formula:

$$PER=\frac{\ \mathrm{weight}\ \mathrm{gain}\ \left(\mathrm{g}\right)\times \mathrm{fish}\ \mathrm{number}\ }{\mathrm{protein}\ \mathrm{intake}\ }$$

### Cumulative mortalities

The abnormal clinical signs in each fish were reported and the mortality rate was analyzed according to Kaplan and Meier [60] to determine the differences among mortalities curve and the postmortem examination of dead fish was recorded.

### Hematological and biochemical analysis

After 30, 45, and 60 days of agrochemical exposure, five fish were randomly selected from each group (15 fish/treatment), anesthetized, and blood samples were collected from caudal vein. A whole blood sample was used for hematologic analyses in tubes containing 10% ethylene-diamine ethylene tetraacetate (EDTA), and hematological values were measured using standard methods. Sahli’s acid haematin method, as described by Zijlstra and Van Kampen [61], was used to calculate hemoglobin (Hb). Neubauer’s improved hemocytometer was used to count red blood cells (RBC) and white blood cells (WBC) using Hyem’s and Turk’s solutions as diluting fluids, as described by Shah and A Altindağ [62]. The W Zijlstra and E Van Kampen [61] microhematocrit method was used to calculate the hematocrit (HCT)/packed cell volume (PCV). The mean corpuscular volume (MCV), mean corpuscular hemoglobin (MCH), and mean corpuscular hemoglobin concentration (MCHC) derived hematological indices were calculated using Lee’s standard formulae [63]. MCV was calculated in femtoliters = PCV/RBC × 10; MCH in picograms = Hb/RBC × 10; and MCHC in milligrams = (Hb in 100 mg blood/PCV) × 100. In extensions, Smears stained with Giemsa / May-Grunwald staining were used for the counts of total numbers of leukocytes (WBC) and thrombocytes by the indirect method described by M Martins, F Pilarsky, E Onaka, D Nomura, J Fenerick, K Ribeiro, D Myiazaki, M Castro and E Malheiros [64]. Differential leukocytic counts (neutrophils, lymphocytes, and monocytes) were determined using an Olympus oil immersion light microscope at 1000 X magnification, and one hundred leukocytes were identified, and the percentage values of different white cells were calculated according to NC Jain [65]. The total number of leukocytes was obtained by subtracting the percentage of thrombocytes from the total of leukocytes plus thrombocytes counted with a Neubauer chamber.

Another blood sample was collected in anticoagulant-free centrifugal tubes and allowed to clot overnight at 4 °C, then were centrifuged at 3000 rpm for 10 min. The non-hemolyzed serum was collected and stored at − 20 °C for further biochemical analysis. Test procedures were performed as per the manufacturer’s instructions (Diamond Diagnostic, Egypt) using 1100 Techocomp UV/visible Spectrophotometer. Total protein (TP) was measured by Weichselbaum’s colorimetric method [66] based on a biuret reaction in an alkaline environment; absorbance photometric measurement with a 550-nm wave based on the method described by CT Weichselbaum [66], A Hubbuch [67]. Albumin was measured using Rodkey’s colorimetric method in the modification of B Doumas, H Biggs, R Arends and P Pinto [68], using bromocresol green in an acidic environment; absorbance photometric measurement with a 600-nm wavelength. The serum globulin (g/dl) level was calculated according to B Doumas, H Biggs, R Arends and P Pinto [68] by mathematical subtraction of albumin value from total protein. The Albumin/Globulin (A/G) ratio was calculated from data on albumin and globulin concentration. Alanine aminotransferase (ALT) and aspartate aminotransferase (AST) were determined calorimetrically according to the method described by S Reitman and S Frankel [69]. The AST and ALT activities were generally assayed by monitoring the concentration of oxaloacetate hydrazine and pyruvate hydrazine, respectively formed with 2, 4-dinitrophenylhydrazine; the colour intensity was measured against the blank at 546 nm and 540 nm, respectively. Creatinine and urea (mg/dl) were determined by Berthelot method [70]. Creatinine reacts with picric acid under the alkaline condition to form a yellow-red complex. The absorbance of the color produced is measured at a wavelength of 505 nm, which is directly proportional to the creatinine content in the sample. Urea was determined by the enzymatic UV kinetic method (Urease–modified Berthelot reaction). Urea is hydrolyzed in the presence of water and urease to produce ammonia and carbon dioxide. The liberated ammonia reacts with a-ketoglutarate in the presence of NADH to yield glutamate. An equimolar quantity of NADH undergoes oxidation during the reaction resulting in a decrease in absorbance which is read at 340 nm that is directly proportional to the urea nitrogen concentration in the sample.

### Oxidative stress biomarkers analysis in livers and kidneys

Three fish were randomly collected from each group and euthanized then their livers and kidneys were homogenized (10% w/v) in 0.1 M Tris HCl buffer (pH 7.4) at 4 °C and centrifuged at 11,000 rpm for 30 min, to extract post mitochondrial supernatant (PMS), and the supernatant was used to determine enzyme and lipid peroxides. The malondialdehyde (MDA) of homogenates was measured immediately, the rest of the homogenates were stored at − 20 °C until tissue superoxide dismutase (SOD) and glutathione peroxidase (GPx) were performed. LPO was estimated by a TBARS (thiobarbituric acid-reactive substances) assay, performed by malondialdehyde (MDA) reaction with 2-thiobarbituric acid (TBA) using the JA Buege and SD Aust [71] method and optical density were measured at 532 nm. SOD activity was measured according to M Paya, B Halliwell and JR Hoult [72]. SOD estimation was based on the generation of superoxide radicals produced by xanthine and xanthine oxidase, which react with 2-(4-iodophenyl)-3(4-nitrophenol)-5phenyltetra-zolium chloride to form a red formazan dye. The SOD activity was then measured at 560 nm and constant temperature (25 °C) by considering the degree of inhibition of this reaction. GPx activity was measured using 2.5, dithiobis-tetranitrobenzoic acid (DTNB) reagent that was measured at 412 nm, according to the modified Mills’ procedure published by DG Hafeman, RA Sunde and WG Hoekstra [73]. All parameters were estimated from homogenate by measuring optical density using 1100 Techocomp UV/visible Spectrophotometer. All the measurements were made in duplicate.

### Statistical analysis

The collected data were subjected to statistical analysis using the SPSS version 22 software computer program, which is available in New York, USA (Inc., 1989–2013). The Pearson correlation coefficient was calculated as a correlation matrix in the form of a rectangular array of integers, which yields the correlation coefficient between the variables. To describe the major relationship between the observed parameters and the chemical exposure, principal components analysis (PCA) was used. A one-way analysis of variance (ANOVA) test with the least significant difference (LSD) technique was used to determine the mean differences. The half-life was determined using probit analysis. To investigate the differences in mortality curves, the Kaplan-Meier test was used.

## Results

### Water analysis

The physiochemical characteristics (DO, pH, temperature, NH_3_, NO_3_, alkalinity, total hardness, and chloride) of different water samples in the aquariums of the experimental fish groups (Table 1) were within acceptable limits published before, which indicated that there was no stress condition correlated to water parameters and the main effects could be attributed to agrochemicals used and possible effects of organic selenium.

**Table 1:** Physicochemical parameters (mean ± SE) of water in experimental *Oreochromis niloticus* groups

Parameters	DO (mg L^− 1^)	pH	Temp	NH_3_ (mg L^− 1^)	NO_3_ (mg L^− 1^)	Hardness (mg L^− 1^)	Cl (mg L^− 1^)	Alkalinity (mg L^− 1^CaCo^3^)
G1	5.76 ± 0.08	8.64 ± 0.28	22.10 ± 0.32	0.038 ± 0.006	2.45 ± 0.68	158.01 ± 19.42	54.74 ± 1.58	26.76 ± 4.94
G2	5.86 ± 0.04	8.88 ± 0.04	22.05 ± 0.65	0.038 ± 0.003	3.27 ± 0.34	180.99 ± 9.71	56.62 ± 0.79	32.61 ± 2.47
G3	5.87 ± 0.05	8.54 ± 0.26	22.06 ± 0.32	0.034 ± 0.005	2.21 ± 0.63	151.13 ± 17.67	54.18 ± 1.44	25.00 ± 4.49
G4	5.88 ± 0.04	8.93 ± 0.04	22.28 ± 0.59	0.040 ± 0.004	3.39 ± 0.31	184.43 ± 8.84	56.90 ± 0.72	33.49 ± 2.24
G5	5.72 ± 0.07	8.52 ± 0.25	22.14 ± 0.30	0.033 ± 0.005	2.15 ± 0.60	149.41 ± 16.95	54.04 ± 1.38	24.57 ± 4.31
G6	5.89 ± 0.03	8.92 ± 0.03	22.34 ± 0.57	0.038 ± 0.006	3.42 ± 0.30	185.29 ± 8.47	56.97 ± 0.69	33.71 ± 2.15
G7	5.80 ± 0.02	8.78 ± 0.07	22.30 ± 0.75	0.036 ± 0.003	2.72 ± 0.19	165.31 ± 5.25	55.34 ± 0.43	28.62 ± 1.33
Permissible limits	≥5 (Lioyd 1992) 3–5 (Anita and Pooja 2013)	6–9 (Popma and Masser, 1999)	11–42 ° C (FAO, 2012)	0.05 (Lawson, 1995) 0.1 max. Tolerable level (Pillay and Kutty, 2005)	≤ 10 (Pillay and Kutty, 2005)	20–300 (Santhosh and Singh, 2007)	60 (Anita and Pooja, 2013)	5–500 (Lawson, 1995)

Means at the same row are not statistically different

**G1** (negative control); **G2** (2 mg L^− 1^ glyphosate); **G3** (0.5 malathion mg L^− 1^); **G4:** (glyphosate 1.6 mg L^− 1^and malathion 0.3 L^− 1^). **G5**: (glyphosate 2 mg L^− 1^and SY 3.3 mg kg ^− 1^), **G6:** (malathion 0.5 mgL^− 1^and SY 3.3 mg kg ^−1^) and **G7:** (glyphosate 1.6 mgL^− 1^; malathion 0.3 mgL^− 1^and SY 3.3 mg kg ^− 1^)**. SY:** selenium yeast

### Growth parameters, feeding efficiency, and cumulative mortalities

**Fig. 1:**
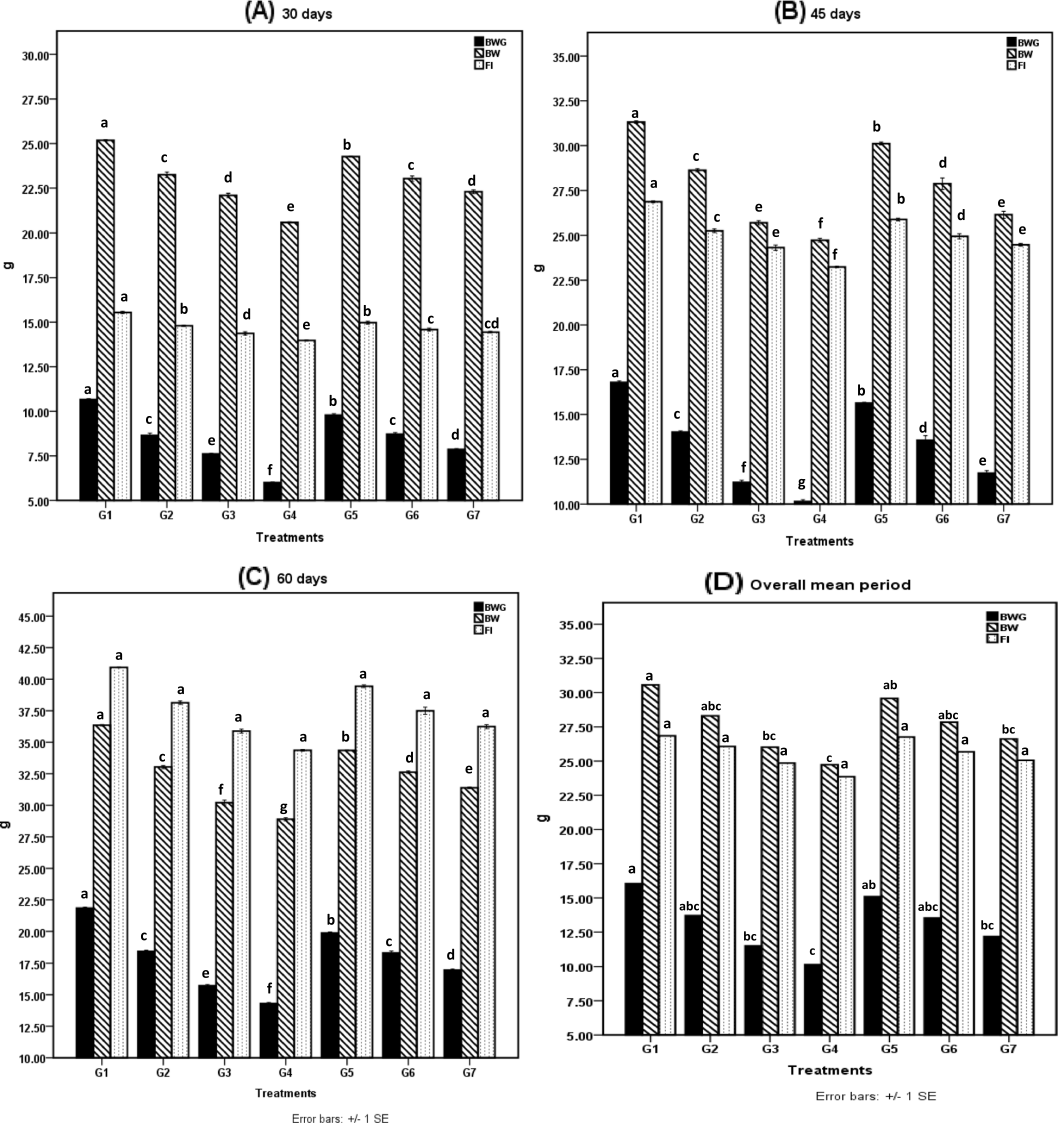
Body weight gain (BWG); body weight (BW) and Feed intake (FI) among the different treated groups during the different experimental periods. Means with different superscripts are statistically different at p ≤ 0.001(BWG) and at p ≤ 0.05 (BW). **A** 30th day of experiment; **B** 45th day of experiment; **C** 60th day of experiment; **D** Average mean

**Fig. 2:**
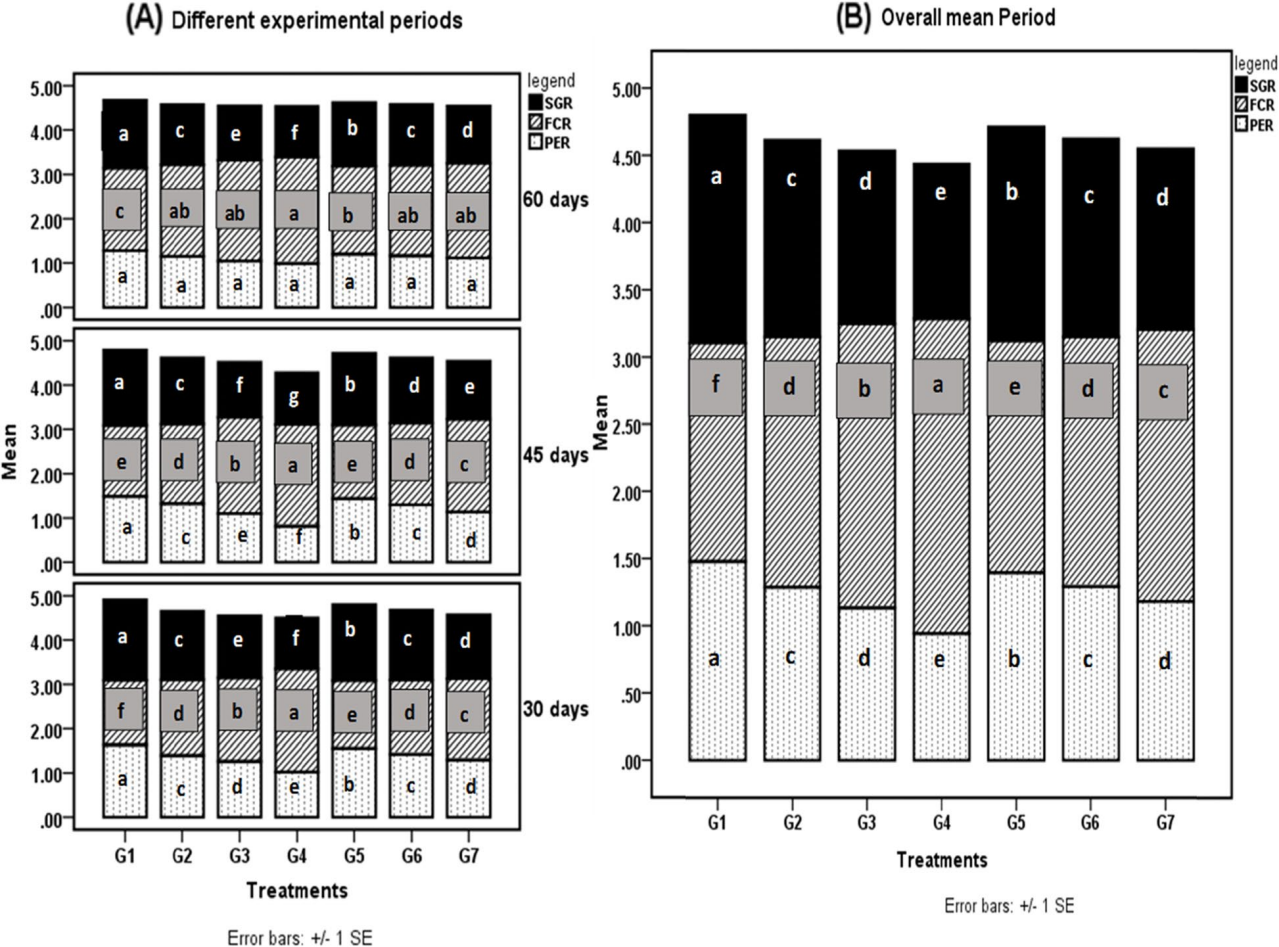
Specific Growth Rate (SGR); Feed Conversion Ratio (FCR) and Protein Efficiency Ratio (PER) among the different treated groups during the different experimental periods. Means with different superscripts are statistically different at *p* ≤ 0.001and at *p* ≤ 0.05 (FCR during 60th day). **A** different experimental periods; **B** Average mean

**Fig. 3:**
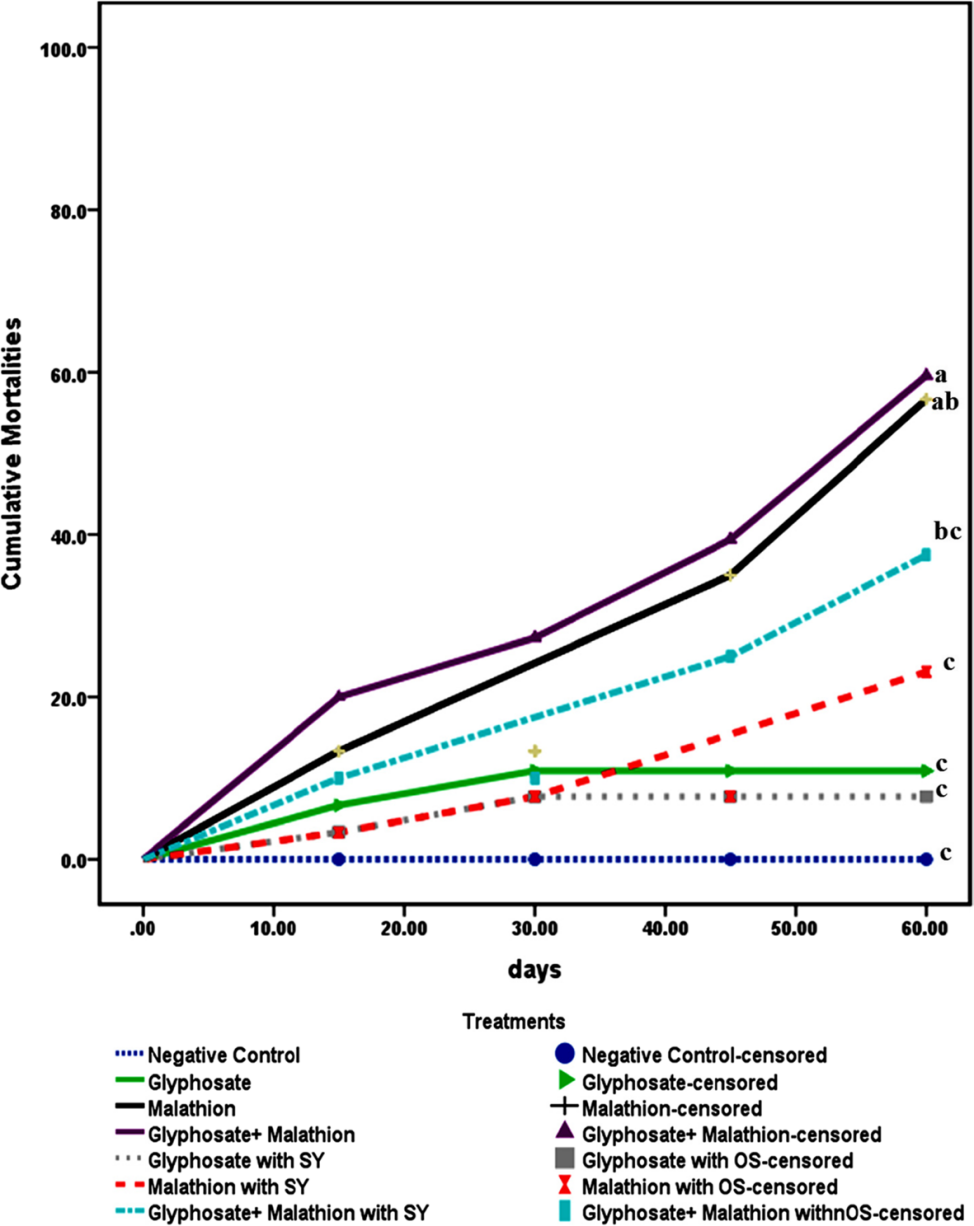
Cumulative mortalities among different treatments during the experimental period. Means with different superscripts are statistically different (P ≤ 0.0001)

Figures 1 and 2 summarize the effects of the investigated agrochemicals on growth parameters and feeding efficiency (BW, BWG, FI, SGR, PER, and FCR). The synergistic effect of the agrochemicals represented a significant (*P* ≤ 0.05) impairment on the examined parameters, followed by malathion then glyphosate exposed groups as compared to control during the different evaluation periods. The enhancement effect of organic selenium supplementation was demonstrated in the pattern of a significant (*P* ≤ 0.05) improvement in the measured parameters compared to chemically stressed groups, and this effect was observed from the first 30 days of the trial. It was worth to report that cumulative mortality rate (Fig. 3) was described in a descending manner as following: G4: 40% (*n* = 12), G3: > 30% (*n* = 9), G7: > 20% (*n* = 6), G2 and G6: > 10% (*n* = 6), G5: > 6.7% (*n* = 2).

### Hematological and biochemical parameters

Results shown are in Table 2 demonstrated alterations in almost all hematological parameters during the different evaluation points along the experimental periods. There was a significant decrease (*P* ≤ 0.05) in erythrocyte count (RBCs) (10^6^/mm^3^), Hb (g dl^− 1^), HCT (%), mean corpuscular hemoglobin concentration (MCHC), mean corpuscular volume (MCV) and mean corpuscular hemoglobin (MCH) as compared to the negative control group all over the different examined dates from experiment beginnings, this reflected a general trend revealing the agrochemicals hazardous effect, which was more pronounced in combined exposure more than the single exposure of each chemical. In the same pattern of agrochemical detrimental effects, fish exhibited pale body and fin (Fig. 5. A) with pale organs, especially the liver (Fig. 5. B). Ameliorative action of SY supplementation was observed in the form of cumulative significant increase (P ≤ 0.05) in the measured parameters as compared to chemically treated groups, and maximum beneficial effect of SY was achieved at 60th day of SY treatment. In the same pattern, blood platelets revealed a significant (*P* ≤ 0.01) thrombocytopenia in fish exposed to glyphosate and/or malathion, which was improved with supplementation of SY. As far as total and differential leucocytes are concerned, our results revealed changes in the leukocyte profile manifested in the form of a significant leukocytosis, neutrophilia, and lymphocytopenia in pesticide exposed groups as compared to control group during the different experimental periods (Table 3**)**.

**Table 2:** Hematological parameters of the experimental *Oreochromis niloticus*

Parameters	Day	G1	G2	G3	G4	G5	G6	G7	Sig. P≤
RBCS (10^6^/mm^3^)	**30**	2.00^a^ ± 0.10	0.26^cd^ ± 0.01	0.22^d^ ± 0.00	0.27^cd^ ± 0.01	1.76^b^ ± 0.04	0.46^c^ ± 0.00	0.43^c^ ± 0.00	**0.01**
	**45**	2.19^a^ ± 0.17	0.88^c^ ± 0.02	1.13^c^ ± 0.01	0.51^c^ ± 0.06	1.37^b^ ± 0.06	1.91^a^ ± 0.05	0.98^c^ ± 0.01	
	**60**	1.90^a^ ± 0.01	0.80^d^ ± 0.03	0.95^c^ ± 0.01	0.55^e^ ± 0.05	1.39^b^ ± 0.02	1.39^b^ ± 0.06	0.98^c^ ± 0.01	**0.05**
	**Average**	2.05^a^ ± 0.08	0.64^cd^ ± 0.07	0.69^cd^ ± 0.11	0.50^d^ ± 0.06	1.50^b^ ± 0.05	1.20^b^ ± 0.16	0.80^c^ ± 0.06	**0.0001**
Hb (g/dl)	**30**	7.38^a^ ± 0.50	2.00^c^ ± 0.03	1.30^de^ ± 0.03	1.13^e^ ± 0.08	4.70^b^ ± 0.06	1.80^cd^ ± 0.01	1.83^cd^ ± 0.01	**0.05**
	**45**	8.50^a^ ± 0.50	2.10^cd^ ± 0.06	2.90^c^ ± 0.23	0.95^d^ ± 0.06	5.50^b^ ± 0.33	4.80^b^ ± 0.30	2.90^c^ ± 0.20	
	**60**	7.50^a^ ± 0.30	2.10^d^ ± 0.06	1.50^e^ ± 0.01	1.36^e^ ± 0.04	5.50^b^ ± 0.09	4.70^c^ ± 0.12	4.70^c^ ± 0.26	**0.01**
	**Average**	7.82^a^ ± 0.29	2.06^d^ ± 0.03	1.82^de^ ± 0.20	1.20^e^ ± 0.14	5.28^b^ ± 0.14	3.84^c^ ± 0.38	3.19^c^ ± 0.34	**0.0001**
HCT %	**30**	23.10^a^ ± 1.61	7.00^c^ ± 0.09	4.90^de^ ± 0.09	4.40^e^ ± 0.24	15.30^b^ ± 0.20	6.60^cd^ ± 0.05	6.50^cd^ ± 0.05	**0.05**
	**45**	26.60^a^ ± 1.60	7.30^cd^ ± 0.19	9.90^c^ ± 0.69	3.80^d^ ± 0.20	17.60^b^ ± 1.00	15.60^b^ ± 0.92	9.98^c^ ± 0.81	
	**60**	23.60^a^ ± 0.90	7.30^d^ ± 0.19	5.64^e^ ± 0.04	5.08^e^ ± 0.14	17.50^b^ ± 0.29	15.20^c^ ± 0.36	15.20^c^ ± 0.79	
	**Average**	24.40^a^ ± 0.80	7.20^d^ ± 0.09	6.40^de^ ± 0.60	4.82^e^ ± 0.40	16.80^b^ ± 0.40	12.50^c^ ± 1.10	10.50^c^ ± 1.00	**0.0001**
MCHC %	**30**	31.80^a^ ± 0.09	28.50^c^ ± 0.06	26.50^d^ ± 0.13	25.60^e^ ± 0.42	31.10^b^ ± 0.02	28.20^c^ ± 0.04	28.20^c^ ± 0.04	**0.01**
	**45**	32.00^a^ ± 0.08	28.70^c^ ± 0.12	29.80^c^ ± 0.21	24.50^d^ ± 0.40	31.40^ab^ ± 0.10	31.10^ab^ ± 0.13	29.80^bc^ ± 0.33	
	**60**	31.90^a^ ± 0.06	28.70^d^ ± 0.12	27.40^e^ ± 0.05	26.70^e^ ± 0.10	31.40^b^ ± 0.03	31.10^c^ ± 0.05	31.10^c^ ± 0.10	**0.05**
	**Average**	31.90^a^ ± 0.04	28.60^c^ ± 0.06	27.60^d^ ± 0.37	25.80^e^ ± 0.40	31.30^a^ ± 0.04	30.10^b^ ± 0.36	29.70^b^ ± 0.30	**0.0001**
MCV (μm^3^)	**30**	116.30^d^ ± 5.60	275.10^b^ ± 17.20	328.00^a^ ± 6.40	159.90^c^ ± 5.90	87.30^e^ ± 3.00	143.80^c^ ± 1.50	149.40^c^ ± 0.80	**0.01**
	**45**	123.80^a^ ± 6.60	83.00^bc^ ± 0.20	87.30^b^ ± 4.50	86.10^c^ ± 16.80	128.50^a^ ± 2.60	83.20^bc^ ± 7.02	100.90^b^ ± 7.86	**0.05**
	**60**	121.10^b^ ± 4.70	90.90^d^ ± 1.35	59.00^e^ ± 0.79	97.20^cd^ ± 10.50	126.50^b^ ± 3.12	111.80^bc^ ± 7.50	155.10^a^ ± 8.40	
	**Average**	120.40^ab^ ± 3.10	149.70^ab^ ± 24.30	162.00^a^ ± 31.9	110.50^b^ ± 10.60	114.10^b^ ± 5.30	112.90^b^ ± 7.35	135.10^ab^ ± 7.40	
MCH (pg)	**30**	37.00^c^ ± 1.70	78.60^b^ ± 5.10	87.10^a^ ± 2.10	41.10^c^ ± 2.20	27.20^d^ ± 0.90	40.60^c^ ± 0.50	42.10^c^ ± 0.20	**0.01**
	**45**	39.70^a^ ± 2.10	23.80^bc^ ± 0.03	26.10^b^ ± 1.50	21.50^c^ ± 4.60	40.30^a^ ± 0.91	30.20^b^ ± 2.60	30.20^b^ ± 2.60	
	**60**	38.60^b^ ± 1.50	26.10^c^ ± 0.20	16.10^d^ ± 0.10	26.10^c^ ± 2.90	39.70^b^ ± 1.01	34.80^b^ ± 2.30	48.30^a^ ± 2.80	
	**Average**	38.40^ab^ ± 1.02	42.80^a^ ± 6.90	43.90^a^ ± 8.34	28.80^b^ ± 2.81	35.70^ab^ ± 1.70	33.80^ab^ ± 1.91	40.20^ab^ ± 2.30	**0.05**
Platelets (10^3^/mm^3^)	**30**	307.40^a^ ± 3.20	167.30^f^ ± 0.70	124.40^g^ ± 1.30	178.50^e^ ± 3.10	243.80^b^ ± 2.30	215.00^d^ ± 4.70	224.40^c^ ± 3.08	
	**45**	331.00^a^ ± 2.90	176.60^d^ ± 3.00	183.90^d^ ± 2.60	158.00^e^ ± 1.10	250.40^b^ ± 4.60	209.20^c^ ± 4.07	250.40^b^ ± 4.40	
	**60**	318.80^a^ ± 1.21	152.80^e^ ± 2.30	134.80^f^ ± 1.20	168.50^d^ ± 6.50	268.30^c^ ± 2.00	283.90^b^ ± 1.60	276.80^bc^ ± 4.00	**0.01**
	**Average**	319.08^a^ ± 2.90	165.60^d^ ± 2.88	146.30^e^ ± 6.64	169.70^d^ ± 3.26	254.20^b^ ± 3.25	236.00^c^ ± 9.29	250.50^bc^ ± 6.08	

Means with different superscripts at the same row are statistically different (P ≤ 0.0001, 0.01 and 0.05)

**G1** (negative control); **G2** (2 mg L^− 1^ glyphosate); **G3** (0.5 malathion mg L^− 1^); **G4:** (glyphosate 1.6 mg L^− 1^and malathion 0.3 L^− 1^). **G5**: (glyphosate 2 mg L^− 1^and SY 3.3 mg kg ^− 1^), **G6:** (malathion 0.5 mgL^− 1^and SY 3.3 mg kg ^− 1^) and **G7:** (glyphosate 1.6 mgL^− 1^; malathion 0.3 mgL^− 1^and SY 3.3 mg kg ^− 1^)**. SY:** selenium yeast

**Table 3:** Total and differential leukocytic count of the experimental *Oreochromis niloticus*

Parameters	Day	G1	G2	G3	G4	G5	G6	G7	Sig. P≤
WBCs (10^3^/mm^3^)	**30**	22.50^c^ ± 0.40	26.70^b^ ± 0.60	32.70^a^ ± 0.87	28.10^b^ ± 0.80	20.9^cd^ ± 0.8	21.8^c^ ± 0.6	19.30^d^ ± 0.40	**0.01**
	**45**	23.60^bc^ ± 0.30	27.30^a^ ± 0.40	23.50^bc^ ± 0.90	26.20^ab^ ± 0.60	22.50^cd^ ± 0.40	23.40^c^ ± 0.40	21.00^d^ ± 0.30	**0.05**
	**60**	22.90^cd^ ± 0.32	28.40^a^ ± 0.37	25.00^b^ ± 0.31	29.60^a^ ± 0.48	21.10^e^ ± 0.33	21.90^de^ ± 0.32	23.40^c^ ± 0.67	
	**Average**	23.00^b^ ± 0.24	27.40^a^ ± 0.32	27.30^a^ ± 1.13	27.70^a^ ± 0.56	21.50^bc^ ± 0.37	22.40^bc^ ± 0.32	21.20^c^ ± 0.53	
Neutrophil%	**30**	27.30^c^ ± 0.30	29.60^bc^ ± 0.90	39.50^a^ ± 2.00	37.90^a^ ± 0.30	28.40^c^ ± 0.40	32.00^b^ ± 0.30	31.50^b^ ± 0.50	
	**45**	26.80^d^ ± 0.60	35.20^a^ ± 0.60	33.40^a^ ± 0.70	34.30^a^ ± 1.20	28.60^cd^ ± 0.10	30.30^bc^ ± 0.40	31.00^b^ ± 0.30	
	**60**	25.30^d^ ± 1.30	29.3^c^ ± 1.10	29.90^c^ ± 0.32	35.50^a^ ± 1.30	28.60^c^ ± 0.18	28.20^c^ ± 0.40	32.50^b^ ± 0.40	**0.01**
	**Average**	26.50^d^ ± 0.51	31.4^b^ ± 0.87	34.60^a^ ± 1.30	35.60^a^ ± 0.66	28.50^c^ ± 0.15	30.20^bc^ ± 0.48	31.70^b^ ± 0.29	**0.05**
Lymphocyte%	**30**	67.80^a^ ± 0.50	44.50^d^ ± 0.60	44.80^d^ ± 0.60	43.00^e^ ± 0.30	59.30^b^ ± 0.40	57.44^c^ ± 0.20	58.90^b^ ± 0.30	
	**45**	67.00^a^ ± 0.31	40.90^e^ ± 0.30	47.30^d^ ± 0.70	39.50^e^ ± 0.40	62.50^bc^ ± 0.60	61.40^c^ ± 0.40	65.20^ab^ ± 0.80	**0.01**
	**60**	67.20^a^ ± 0.40	38.20^d^ ± 1.20	42.80^c^ ± 0.97	36.50^d^ ± 1.32	67.70^a^ ± 0.18	66.20^a^ ± 1.17	59.70^b^ ± 0.40	
	**Average**	67.34^a^ ± 0.25	41.22^d^ ± 0.81	44.57^c^ ± 0.68	40.14^d^ ± 0.97	63.10^b^ ± 0.95	61.70^b^ ± 1.04	61.30^b^ ± 0.80	
Monocyte%	**30**	7.50^c^ ± 0.12	9.00^a^ ± 0.08	7.50^bc^ ± 0.10	7.70^bc^ ± 0.35	8.10^b^ ± 0.11	6.20^d^ ± 0.10	6.50^d^ ± 0.19	**0.05**
	**45**	7.60^b^ ± 0.18	9.30^a^ ± 0.18	6.70^c^ ± 0.18	6.20^cd^ ± 0.30	7.50^b^ ± 0.37	6.30^cd^ ± 0.18	5.94^d^ ± 0.03	
	**60**	7.6^c^ ± 0.18	10.0^e^ ± 0.31	5.7^a^ ± 0.23	8.6^d^ ± 0.18	7.7^c^ ± 0.24	7.50^c^ ± 0.30	6.26^a^ ± 0.35	
	**Average**	7.62^b^ ± 0.09	9.4^a^ ± 0.15	6.6^c^ ± 0.2	7.55^b^ ± 0.31	7.82^b^ ± 0.15	6.70^c^ ± 0.19	6.25^c^ ± 0.14	**0.01**

Means with different superscripts at the same row are statistically different (0.01 and 0.05)

**G1** (negative control); **G2** (2 mg L^− 1^ glyphosate); **G3** (0.5 malathion mg L^− 1^); **G4:** (glyphosate 1.6 mg L^− 1^and malathion 0.3 L^− 1^). **G5**: (glyphosate 2 mg L^− 1^and SY 3.3 mg kg ^− 1^), **G6:** (malathion 0.5 mgL^− 1^and SY 3.3 mg kg ^− 1^) and **G7:** (glyphosate 1.6 mgL^− 1^; malathion 0.3 mgL^− 1^and SY 3.3 mg kg ^− 1^)**. SY:** selenium yeast

A significant (*P* ≤ 0.0001) hypoproteinemia accompanied by a significant decrease in both albumin and globulin concentrations was observed in fish exposed to pesticides on 30th, 45th and 60th days, also malathion was more toxic to fish than glyphosate and had an adverse effect on total serum protein. The results of this study showed a significant increase in ALT and AST activity with creatinine and urea in the blood samples of fish exposed to agrochemicals compared to controls at 30th, 45th, and 60th days of exposure (Tables 4 and 5), with malathion-treated group exhibited the highest AST levels and negatively impacting kidney function by increasing creatinine and urea to the highest significant levels (*P* ≤ 0.05), proving that malathion is more toxic than glyphosate. On the other hand, the addition of SY decreased the hepato-renal toxicity of these chemicals (Tables 4 and 5).

**Table 4:** Serum protein parameters of the experimental *Oreochromis niloticus*

Parameters (g/dL)	Day	G1	G2	G3	G4	G5	G6	G7	Sig. P≤
Total protein	**30**	8.56^a^ ± 0.34	4.94^d^ ± 0.08	3.63^e^ ± 0.31	3.48^e^ ± 0.42	7.72^b^ ± 0.35	8.23^a^ ± 0.17	7.14^c^ ± 0.48	**0.05**
	**45**	9.92^a^ ± 0.58	5.40^d^ ± 0.35	3.40^f^ ± 0.14	4.07^e^ ± 0.26	8.28^b^ ± 0.40	7.98^b^ ± 0.50	6.60^c^ ± 0.24	
	**60**	11.40^a^ ± 0.33	6.20^d^ ± 0.07	4.00^f^ ± 0.29	4.90^e^ ± 0.07	8.76^b^ ± 0.61	9.00^b^ ± 0.38	7.60^c^ ± 0.34	**0.01**
	**Average**	9.97^d^ ± 0.30	5.53^d^ ± 0.10	3.70^e^ ± 0.09	4.15^e^ ± 0.16	8.25^b^ ± 0.10	8.42^b^ ± 0.10	7.12^c^ ± 0.14	**0.0001**
Albumin	**30**	5.2^a^ ± 0.11	3.69^d^ ± 0.30	2.90^e^ ± 0.41	2.08^f^ ± 0.21	3.88^d^ ± 0.25	4.87^b^ ± 0.14	4.48^c^ ± 0.18	**0.05**
	**45**	5.40^a^ ± 0.72	2.89^c^ ± 0.36	1.72^d^ ± 0.15	2.13^d^ ± 0.36	4.97^a^ ± 0.74	4.08^b^ ± 0.18	3.93^d^ ± 0.40	
	**60**	7.04^a^ ± 0.1	3.55^e^ ± 0.4	3.07^f^ ± 0.20	3.82^e^ ± 0.21	5.66^b^ ± 0.47	5.24^c^ ± 0.22	4.90^d^ ± 0.07	**0.01**
	**Average**	5.89^a^ ± 0.24	3.38^c^ ± 0.11	2.56^d^ ± 0. 16	2.67^d^ ± 0.22	4.83^b^ ± 0.23	4.73^b^ ± 0.13	4.44^b^ ± 0.12	
Globulin	**30**	3.34^a^ ± 0.45	1.24^c^ ± 0.22	0.73^d^ ± 0.17	1.40^c^ ± 0.20	3.80^a^ ± 0.50	3.36^a^ ± 0.31	2.66^b^ ± 0.53	**0.05**
	**45**	4.5^a^ ± 0.18	2.5^d^ ± 0.10	1.68^e^ ± 0.18	1.94^e^ ± 0.20	3.30^c^ ± 0.25	3.90^b^ ± 0.51	2.67^d^ ± 0.23	**0.01**
	**60**	4.38^a^ ± 0.21	2.65^c^ ± 0.2	0.97^d^ ± 0.11	1.08^d^ ± 0.19	3.09^c^ ± 0.45	3.81^b^ ± 0.58	2.70^c^ ± 0.31	**0.05**
	**Average**	4.08^a^ ± 0.15	2.15^d^ ± 0.17	1.13^e^ ± 0.11	1.40^e^ ± 0.11	3.41^b^ ± 0.13	3.69^b^ ± 0.13	2.67^c^ ± 0.09	
A: G	**Day 30**	1.60^cd^ ± 0.28	3.12^b^ ± 0.72	4.21^a^ ± 0.81	1.55^cd^ ± 0.34	1.04^b^ ± 0.19	1.47^cd^ ± 0.18	1.79^a^ ± 0.43	**0.01**
	**Day 45**	1.20^bc^ ± 0.19	1.13^c^ ± 0.15	1.04^c^ ± 0.18	1.13^c^ ± 0.27	1.5^a^ ± 0.32	1.07^c^ ± 0.16	1.50^ab^ ± 0.29	**0.05**
	**Day 60**	1.61^cd^ ± 0.06	1.36^d^ ± 0.22	3.19^b^ ± 0.42	3.69^a^ ± 0.75	1.88^c^ ± 0.31	1.42^cd^ ± 0.27	1.71^cd^ ± 0.34	
	**Average**	1.47^d^ ± 0.07	1.87^c^ ± 0.26	2.80^a^ ± 0.37	2.12^b^ ± 0.32	1.48^d^ ± 0.11	1.32^d^ ± 0.06	1.71^cd^ ± 0.08	

Means with different superscripts at the same row are statistically different (P ≤ 0.0001, 0.01 and 0.05)

**G1** (negative control); **G2** (2 mg L^−1^ glyphosate); **G3** (0.5 malathion mg L^−1^); **G4:** (glyphosate 1.6 mg L^−1^and malathion 0.3 L^−1^). **G5**: (glyphosate 2 mg L^−1^and SY 3.3 mg kg ^− 1^), **G6:** (malathion 0.5 mgL^− 1^and SY 3.3 mg kg ^− 1^) and **G7:** (glyphosate 1.6 mgL^− 1^; malathion 0.3 mgL^− 1^and SY 3.3 mg kg ^− 1^)**. SY:** selenium yeast

**Table 5:** Liver enzymes and kidney markers of the experimental *Oreochromis niloticus*

Parameters	Day	G1	G2	G3	G4	G5	G6	G7	Sig. P≤
ALT (u/L)	**30**	37.00^d^ ± 0.30	73.30^c^ ± 1.64	80.10^a^ ± 0.81	72.60^b^ ± 1.45	57.60^b^ ± 2.18	40.20^d^ ± 0.24	58.10^c^ ± 2.42	**0.01**
	**45**	37.80^f^ ± 0.64	64.50^d^ ± 2.26	87.0^a^ ± 0.63	80.40^b^ ± 0.49	60.62^c^ ± 1.60	37.60^f^ ± 0.48	49.50^e^ ± 0.49	**0.05**
	**60**	39.06^f^ ± 0.32	56.40^c^ ± 0.81	87.4^a^ ± 0.67	76.00^b^ ± 1.30	51.10^d^ ± 0.98	38.40^f^ ± 0.67	47.80^e^ ± 0.64	**0.001**
	**Average**	38.00^f^ ± 0.32	63.02^c^ ± 2.60	84.8^a^ ± 0.97	76.30^b^ ± 1.05	58.20^d^ ± 0.90	38.70^f^ ± 0.39	51.80^e^ ± 1.40	**0.05**
AST (u/L)	**30**	40.60^e^ ± 0.18	45.10^cd^ ± 0.84	71.0^a^ ± 1.14	50.60^b^ ± 1.45	46.10^c^ ± 0.81	41.70^e^ ± 0.24	42.70^de^ ± 0.65	
	**45**	35.20^f^ ± 0.49	47.40^c^ ± 1.31	67.3^a^ ± 0.48	55.90^b^ ± 1.45	38.80^de^ ± 0.96	37.20^ef^ ± 0.60	40.80^d^ ± 0.86	
	**60**	38.20^f^ ± 0.18	44.30^c^ ± 0.18	67.0^a^ ± 1.62	59.60^b^ ± 1.64	37.80^de^ ± 0.84	36.90^ef^ ± 0.32	37.80^d^ ± 0.86	**0.0001**
	**Average**	38.00^e^ ± 0.61	45.60^c^ ± 0.59	68.4^a^ ± 0.79	55.40^b^ ± 1.28	40.90^d^ ± 1.09	38.60^de^ ± 0.64	40.40^de^ ± 0.68	**0.05**
Creatinine (mg/dl)	**30**	0.72^e^ ± 0.03	1.60^b^ ± 0.07	1.77^ab^ ± 0.02	1.83^a^ ± 0.04	1.30^c^ ± 0.03	1.10^d^ ± 0.06	1.32^c^ ± 0.24	**0.001**
	**45**	0.71^d^ ± 0.06	1.71^b^ ± 0.08	1.60^b^ ± 0.04	2.04^a^ ± 0.05	1.10^c^ ± 0.06	0.90^cd^ ± 0.03	1.11^c^ ± 0.04	
	**60**	0.94^c^ ± 0.06	1.47^a^ ± 0.05	2.05^a^ ± 0.13	1.87^a^ ± 0.05	1.27^b^ ± 0.06	0.90^c^ ± 0.03	1.18^b^ ± 0.19	
	**Average**	0.80^e^ ± 0.04	1.78^b^ ± 0.05	1.82^ab^ ± 0.06	1.91^ab^ ± 0.03	1.22^c^ ± 0.03	0.96^d^ ± 0.03	1.20^c^ ± 0.02	**0.05**
Urea (mg/dl)	**30**	3.70^e^ ± 0.06	5.54^c^ ± 0.24	8.01^a^ ± 0.08	6.62^b^ ± 0.04	5.36^c^ ± 0.13	4.17^d^ ± 0.19	5.59^c^ ± 0.16	**0.001**
	**45**	3.41^f^ ± 0.06	5.67^c^ ± 0.14	7.19^a^ ± 0.17	6.82^b^ ± 0.11	4.00^e^ ± 0.06	3.97^e^ ± 0.11	4.92^d^ ± 0.06	
	**60**	3.52^f^ ± 0.09	6.19^c^ ± 0.14	7.46^b^ ± 0.079	7.81^a^ ± 0.08	5.23^d^ ± 0.153	3.78^f^ ± 0.096	4.83^c^ ± 0.142	
	**Average**	3.54^f^ ± 0.05	5.80^c^ ± 0.12	7.5^ab^ ± 0.11	7.08^b^ ± 0.14	4.8^d^ ± 0.17	3.97^e^ ± 0.08	5.10^d^ ± 0.11	**0.01**

Means with different superscripts at the same row are statistically different (P ≤ 0.0001, 0.001, 0.01 and 0.05)

**G1** (negative control); **G2** (2 mg L^−1^ glyphosate); **G3** (0.5 malathion mg L^−1^); **G4:** (glyphosate 1.6 mg L^−1^and malathion 0.3 L^−1^). **G5**: (glyphosate 2 mg L^−1^and SY 3.3 mg kg ^−1^), **G6:** (malathion 0.5 mgL^− 1^and SY 3.3 mg kg ^− 1^) and **G7:** (glyphosate 1.6 mgL^− 1^; malathion 0.3 mgL^− 1^and SY 3.3 mg kg ^− 1^)**. SY:** selenium yeast

### Principal component analysis (PCA) between the different variables

**Fig. 4:**
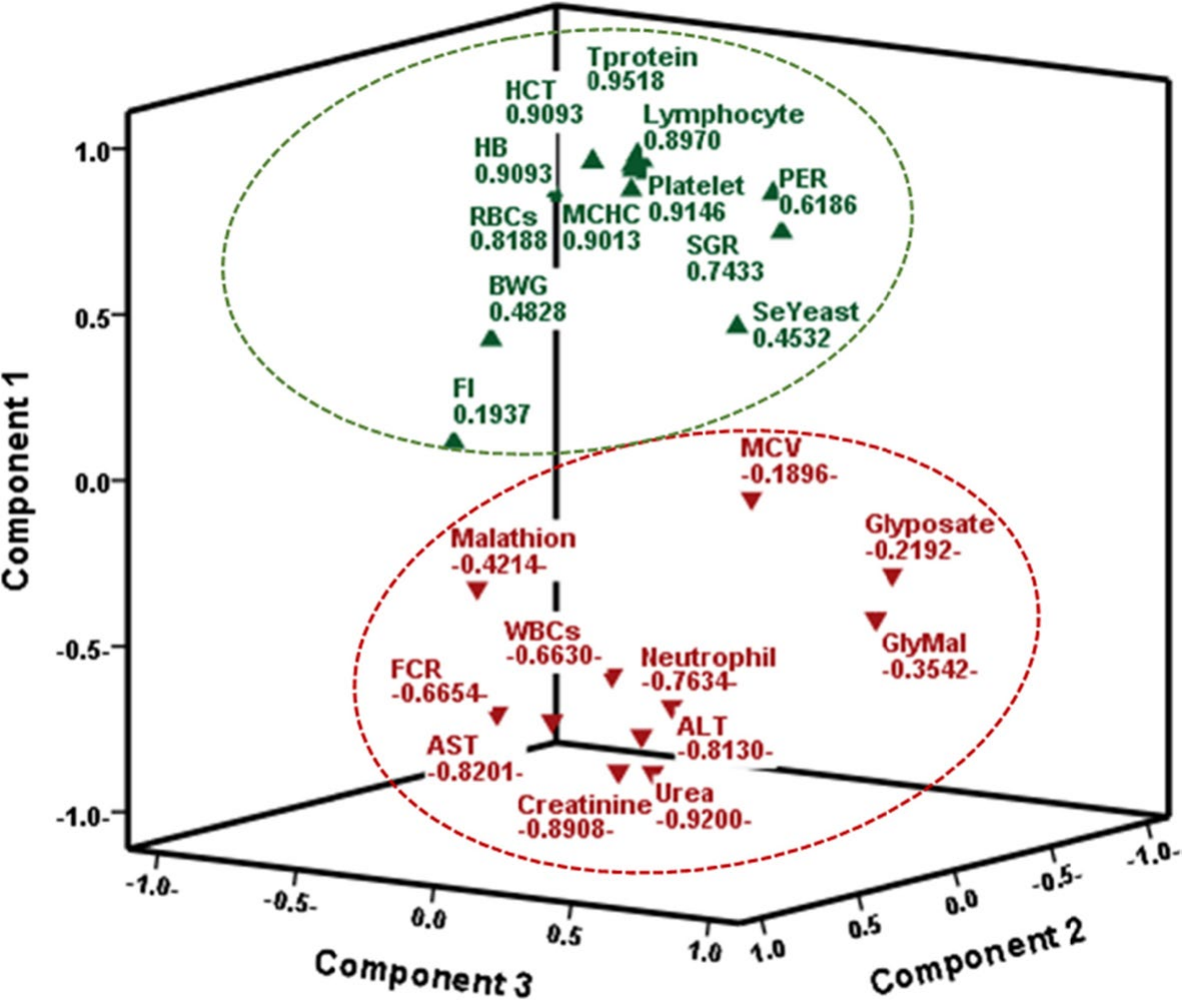
Principle component analysis of Glyphosate and /or Malathion intoxication with hematological; biochemical and growth performance parameters in *Oreochromis niloticus*. Data were extracted by principal component analysis (PCA) and rotated through Varimax with Kaiser Normalization. Explained variance % was 53.73, 12.35, 10.1, 7.6 and 4.7% while Cumulative % was 53.73, 66.1 and 76.16, 83.8 and 88.5% for components (PC1, PC2, PC3, PC4 and PC5, respectively). Positive loading values in PC1 were represented by green upward arrows, while the red downward arrows represented negative loading

Principal component analysis (PCA) was used to estimate the distribution pattern of the individual association of parameters with significant correlations divided into components. After the verification of the data’s validity by Bartlett’s sphericity test (< 0.001) and KMO test for the measured parameters, the parameters produced three principal components (PC) (eigenvalues > 1) explaining the total variances of 88.5%. Corresponding, variable loadings and explained variance are presented in Fig. 4**.** PC1 explained 53.74% variance and negative loadings of agrochemicals doses were reported as malathion>combined > glyphosate exposure, and they were directly correlated with increased urea> creatinine> AST > ALT> FCR > WBCs> A/G, and they have also inversely correlated with the other examined parameters in a declining level of them. On the contrary, SY supplementation was correlated in a positive loading directly with total protein > platelet count > globulin > RBCs > Hb, HCT, % and lymphocytes > albumin > SGR > PER > BWG while inversely associated with the other parameters, confirming its corrective role.

**Fig. 5:**
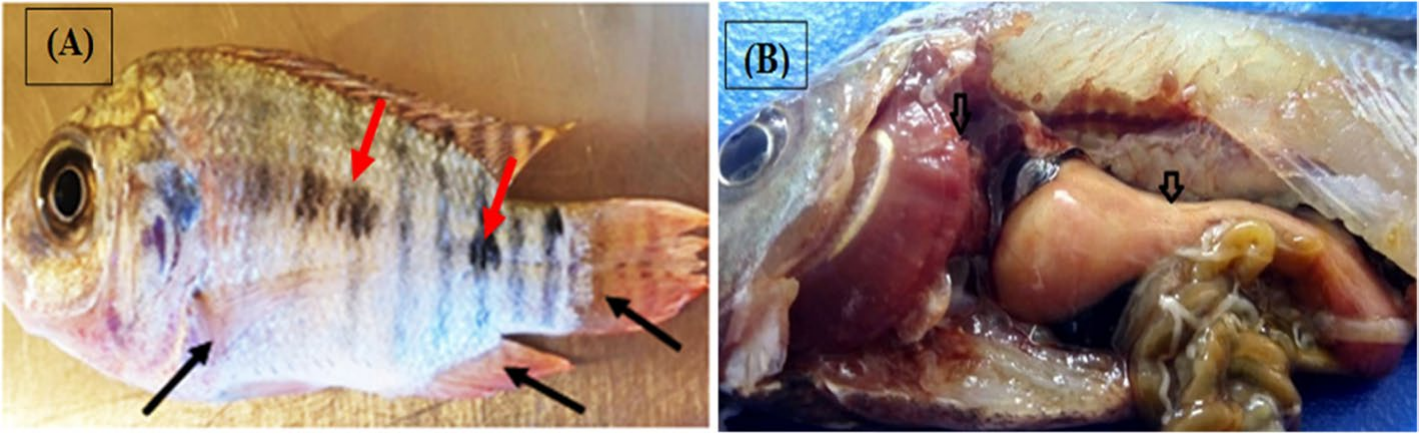
Signs of anemia in groups exposed to agrochemicals. **A** Black arrows indicated anemic pale fish with pale fins due to reduction in RBCS and Hb content., red arrows represent dark pigmentation. **B** Postmortem examination showed pale liver and congested gall bladder

### Oxidative stress biomarkers analysis in livers and kidneys

Table 6 shows that fish exposed to malathion had a strong positive correlation with MDA, SOD, and GPX in both tissues, while the glyphosate-exposed fish showed a positive correlation that was significant in liver MDA. Fish exposed to a combination of agrochemicals showed a highly significant correlation with liver (MDA and SOD) and kidney GPX. The corrective role of SY, on the other hand, was shown by a highly significant negative correlation with all measurable oxidative stress biomarkers.

**Table 6:** Correlation coefficient between agrochemicals’ exposure, Selenium Yeast supplementation and oxidative stress markers in *Oreochromis niloticus*

Parameters		MDA (nmol/g)		SOD (mg/g)		GPx (nmol/g)	
		Liver	Kidney	Liver	Kidney	Liver	Kidney
**Glyphosate**		0.227^*^	0.037	0.156	0.072	0.112	0.170
**Malathion**		0.360^**^	0.290^**^	0.322^**^	0.193^*^	.253^**^	0.272^**^
**Glyphosate and malathion**		0.345^**^	0.116	0.257^**^	0.130	0.190	0.259^**^
**SY**		−0.474^**^	−0.558^**^	−0.578^**^	−0.643^**^	−0.615^**^	−0.579^**^
**MDA (nmol/g)**	**Liver**	1	0.749^**^	0.895^**^	0.718^**^	0.872^**^	0.891^**^
	**Kidney**		1	0.765^**^	0.928^**^	0.895^**^	0.794^**^
**SOD (mg/g)**	**Liver**			1	0.743^**^	0.890^**^	0.893^**^
	**Kidney**				1	0.851^**^	0.766^**^
**GPX kidney (nmol/g)**	**Liver**					1	0.926^**^
	**Kidney**						1

*Correlation is significant at the 0.05 level (2-tailed), **. Correlation is significant at the 0.01 level (2-tailed)

**SY:** selenium yeast**.** Malondialdehyde (MDA), superoxide dismutase (SOD) and glutathione peroxidase (GPx)

## Discussion

All results for DO, pH, Temperature, ammonia, alkalinity, chloride were within acceptable limits published before, whereas DO was higher than 5 mg/L in all experimental groups, this result agreed with described limits by R Lloyd [74] and A Bhatnagar and P Devi [75]. Also, pH and temperature showed averages ranging between 8 and 20 °C, respectively. These levels are within limits for growing tilapia. Levels of nitrogenous compounds: NH_3_ and NO_3_ lied within acceptable limits recommended by TB Lawson [76], TVR Pillay and MN Kutty [77], respectively. At the same time, hardness levels of chloride and alkalinity were within acceptable limits described by A Bhatnagar and P Devi [75], TB Lawson [76], B Santhosh and N Singh [78]. Our findings revealed that there was no stress condition correlated to measured water parameters, this revealed that the main effects could be attributed to agrochemicals used and their mitigation by using SY.

Concerning the adverse effects of glyphosate on *Oreochromis niloticus* growth performance, the results were consistent with those obtained by PC Giaquinto, MB de Sá, VS Sugihara, BB Gonçalves, HC Delício and A Barki [79]. The latter authors observed that glyphosate-based herbicide at sublethal concentrations (1.8 ppm) affected feed intake in *pacu* fish and thus, inhibited its growth. Acute exposure of salmon to 1 ppm or higher of glyphosate resulted in reduced electro-olfactogram activity when it detected L-serine in the environment, glyphosate by its role resembles the amino acid glycine and there is some overlapping for the same active site for L-serine substance and the fish could not detect L-serine or respond to its presence [80]. Our findings are in agreement with those of UA Muhammad, NA Yasid, HM Daud and MY Shukor [57], who reported a negative correlation between glyphosate concentration and toxicity parameters such as specific growth rate (SGR).

Generally, it was found that pollutants affected specific processes associated with bioenergetics, such as feeding, assimilation, excretion, and metabolism so delayed fish growth [81]. Furthermore, our result agreed with those reported by CA Laetz, DH Baldwin, TK Collier, V Hebert, JD Stark and NL Scholz [82] who indicated that histopathological damage to the liver, pancreas, and intestine may result in decreased feed digestion and metabolism efficiency because these tissues play critical roles in the regulation of biochemical parameters, particularly proteins, lipids, carbohydrates, and hormones, as well as the synthesis and secretion of digestive enzymes. Other important factors that explain the delay in fish growth could be the transformation into the energy of a portion of nutrients from the digestion of feed consumed to cope with chemical stress that constitutes the exposure to agricultural pesticides [83]**.** The highest reduction in growth in the combined agrochemical exposed group may be attributed to a synergistic impact between glyphosate and malathion; this effect could be explained in light of the fact that binary pesticide combinations generated synergistic acetyl-cholinesterase inhibition [82].

In the current study, there was a positive correlation between SY supplementation and the measured growth performance parameters that indicated improvements in the measured parameters accompanied by decreased cumulative mortalities in both the SY which was in some parameters very close to the non-treated control group. These results assured the fundamental usage of organic SY compounds as a protective antioxidant material to reduce the toxic effects of pesticides [84]. Generally, under normal culture conditions, M Abdel-Tawwab and M Wafeek [85] reported that feed supplemented with 5.54 mg kg^− 1^ improved growth in tilapia. Similarly, SBd Fonseca, JHVd Silva, EM Beltrão Filho, PdP Mendes, JBK Fernandes, ALL Amancio, J Jordão Filho, PBd Lacerda, FRPdJFS Silva and Technology [86] demonstrated that the diet containing 0.2 mg kg^− 1^ organic selenium produced weight gain, length gain, and feed conversion ratios comparable to the treatment containing 0.4 mg kg^− 1^ inorganic selenium. The authors previously mentioned attributing the improvement in growth performance parameters to an increase in glutathione peroxidase concentrations in the blood of tilapia-fed selenium in the diet. Furthermore, S Iqbal, U Atique, M Mughal, N Khan, M Haider, K Iqbal and M Akmal Rana [87] stated that supplementing selenium (2 mg kg^− 1^) in tilapia feed promotes better physiological performance and productivity, thereby enhancing fish growth and paving the way for an increased supply of selenium-fortified fish meat. Dietary organic Se incorporation at 0.45 mg Kg^− 1^ provided satisfactory results in various growth parameters and was an effective supplement in salmonid fish diets [88]**.** Similarly, A El-Kader, F Marwa, AF Fath El-Bab, MF Abd-Elghany, A-WA Abdel-Warith, EM Younis and MAJBTER Dawood [89] recommended Se nanoparticles at the rate of 0.5–1 mg kg^− 1^ diet to maintain the optimal growth performance of European seabass (*Dicentrarchus labrax*). Additionally, M Naiel, S Negm, S El-hameed and H Abdel-Latif [90] exemplified that dietary inclusion with 0.36–0.39 mg OS kg^− 1^ diet improved the growth, immunity and modulated the stress responses in Nile tilapia reared under sub-optimal temperature. Moreover, S Ghaniem, E Nassef, AI Zaineldin, A Bakr and S Hegazi [91] investigated the effects of different sources of selenium of 1 mg kg^− 1^ diet (inorganic (SSE), organic (OSE), and elemental nano-selenium (NSE)) on the performance *Oreochromis niloticus* and found that dietary selenium supplementation significantly improved growth performance parameters (*P* < 0.05), with the highest values recorded in the OSE supplemented and control groups.

Severe anemia with marked thrombocytopenia was observed in fish exposed to glyphosate or malathion, as evidenced by significant reductions in RBCs (10^6^/mm^3^), Hb (g/dl), MCHC %, and HCT (%) with a significant increase in MCV and MCH levels. The previous findings were confirmed by the inverse relationship between pesticide exposure and RBCs, Hb, and HCT values obtained by PCA, and these alterations were increased as the exposure period increased. Glyphosate-contaminated water contributes to changes in blood cell parameters [92]. This could be due to cell destruction and/or a decrease in cell volume because of the negative effects of pesticides [93]. Alteration in blood indices was directly associated with concentration and exposure period of malathion, and this reduction could be attributed to the effect on gills, anda decrease in available oxygen, as well as hemolysis [94]. Furthermore, these effects may be a result of deleterious effects on the hematopoietic organs, reducing the supply of RBCs through decreased production and/or an increased rate of removal from the circulatory system. Besides, the decrease in Hb level may be due to the toxic effects of malathion and glyphosate on the synthesis of this molecule, which may also disrupt it by affecting the activity of enzymes involved in the synthesis. Therefore, the detected anemia could be related to erythrocyte inhibition, hematopoiesis, osmotic dysregulation, or an increased rate of red blood cell destruction in the hematopoietic organ [95, 96]. The reduction in MCHC was probably characterized by an increase in the generation and secretion of reticulocytes, which were larger in size but contained less Hb than mature red blood cells [97]. In addition, SJ Gholami-Seyedkolaei, A Mirvaghefi, H Farahmand and AA Kosari [98] indicated that the increase in the number of immature RBCs could lead to increased values of MCV and MCH indices.

The protective role of SY supplementation in preventing anemia in fish exposed to pesticides and herbicides might be supported by the positive correlation between SY in diet and RBCs and Hb level in the present study. That could be attributed to the fact that Se increases the stability of the RBCs and thrombocyte membranes and their survivability by protecting them against oxygen-free radicals, causing membrane damage, cell hemolysis, and thrombocytopenia. Se with a concentration of 0.7 mg kg^− 1^ of feed had the ability to protect the fish cell against oxidation due to chemical pollution [99]. Additionally, previous results showed similar enhancements in the Hb, RBCs, and PCV indices with dietary Se in common carp [100]. Also, A El-Kader, F Marwa, AF Fath El-Bab, MF Abd-Elghany, A-WA Abdel-Warith, EM Younis and MAJBTER Dawood [89] reported significantly higher values of Hb, PCV, RBCs, and WBCs in fish fed 0.5—1 mg kg^− 1^ Se nanoparticles. In the same context, S Ghaniem, E Nassef, AI Zaineldin, A Bakr and S Hegazi [91] reported that the selenium-supplemented groups had the highest packed-cell volume, hemoglobin, and red blood cell levels, with the highest values seen in the control group (*P*< 0.05).

WBCs and PCA revealed leukocytosis which could be attributed to the fact that when water quality is altered by toxic substances, leukocytosis occurs as a normal physiological response of fish to foreign substances that assisted in the elimination of cell debris and necrotic tissue and stimulate immune defense [101]. A significant increase in WBCs in common carp following glyphosate exposure was attributed to the immune-toxic effects with changes in glyphosate-caused cytokines that may lead to immune suppression or excessive activation in the treated groups as well as immune dysfunction or reduced immunity [98]. The significant increase in WBCs count in the current study indicated hypersensitivity of leucocytes to malathion and glyphosate. These changes could be due to immunological reactions to produce antibodies in response to stress caused by organophosphorus pesticides [23]. Simultaneously, the leukocytosis observed probably reflected the increased leukocytic demand for the removal of cellular debris at a faster rate [102]. Furthermore, in the current study, the lymphocytopenia and neutrophilia are supported by the findings of SJ Gholami-Seyedkolaei, A Mirvaghefi, H Farahmand and AA Kosari [98] who explained the response as it could be considered as clear responses by the fish during exposure to a wide range of toxicants.

The beneficial effect of SY supplementation on WBCs was illustrated as a non-significant difference between the negative control group and SY-treated groups for most of the experimental periods, additionally, a negative association between SY supplementation and WBCs was recorded. Our results were consistent with HS Hamed [23] who noted that the use of Se in the diet was an effective way to counteract the toxicity of malathion in tilapia fish and recommended the use of Se as a protective dietary supplement against malathion-induced toxicity to improve fish health.

There was a negative relationship between pesticide exposure and blood proteins. Whereas, the correlation with liver enzymes and kidney markers was positive and this could be attributed to impaired albumin synthesis as in chronic hepatic insufficiency or hepatitis and chronic renal affections**,** as well as excessive protein loss due to alterations of necrosis in the kidney or hepatocytes destruction in the toxicity of organophosphates and the consequent impairment of protein synthesis [103, 104]. Moreover, the reduction in albumin and globulin levels could be a consequence of a decrease in blood viscosity or a decrease in body immunity because of the liver’s inability to synthesize enough of them. Longer periods of herbicide exposure would be expected to trigger enough damage to mitochondrial membranes to release AST into the blood, furthermore, the activities of ALT and/or AST are well-known as stress bio-indicators of hepatotoxicity and liver, gill, and kidney damage [105].

Results were consistent with those of SJ Gholami-Seyedkolaei, A Mirvaghefi, H Farahmand and AA Kosari [98], who concluded that the activity of renal and hepatic AST and ALT in glyphosate-treated groups was significantly higher than the control group at different experimental periods. Regarding glyphosate’s toxic effect on the kidney, our results were in agreement with those of S Ayoola [21] who recorded a deleterious toxic effect of glyphosate on the renal function of *Oreochromis niloticus* that showed a great susceptibility to herbicides.

Our findings indicate that chronic malathion intoxication with a sub-lethal dose had the greatest impact on the fish, which could be explained by the fact that structural and soluble proteins were found to be decreased because of high proteolytic activity and inefficiency in protein biosynthesis following malathion exposure [106], which consequently decreases the protein content confirming the intoxication caused by malathion [107, 108]**.** There were also significant increases in creatinine and urea levels following acute malathion exposure, indicating that malathion had an adverse effect on the kidneys [109]. The results obtained are partially consistent with those obtained by R Magar and A Shaikh [110], who found the influence of malathion on damaging organs such as the kidney and liver in the fish *Channa punctatus,* exposed to sub-lethal quantities of malathion for a subsequent 7 days.

The current study’s increased oxidative stress markers in fish exposed to agrochemicals are the result of oxidative damage and a reduction in antioxidant defense, and these findings have been confirmed by Awasthi Y, Ratn A, Prasad R, Kumar M and  Trivedi S [111]. MDA increased significantly because of oxidative stress [112]. Our investigation concluded that greater ROS generation altered the elevation of SOD and GPx. The fish’s defensive strategy was to fight, eliminate, or neutralize the damaging effects of ROS and protect the system from oxidative stress [113].

In the present study, the addition of SY showed significant improvements in *Oreochromis niloticus* blood proteins, liver enzymes, and kidney markers compared to pesticide groups during different evaluated periods of agrochemicals exposure. These findings could be strengthened by the inverse relationship between SY supplementation and the previously mentioned parameters. This is consistent with other findings in our study proving that Se has hepatoprotective properties against organophosphorus pesticides and heavy metals that induce liver damage [114, 115] and its ability to protect the host cell against oxidation due to environmental challenges, with an optimum level between 0.15 and 0.8 mg/kg diet [99]. In the same context, M Abdel-Tawwab and M Wafeek [85] discovered that tilapia diets enriched with 0.54 mg kg^− 1^ organic Se reduced the adverse effects of pesticide stress. Furthermore, MA Naiel, A Nasr and M Ahmed [116] concluded that organic Se 0.6 mg kg^− 1^ supplementation decreased serum creatinine and uric acid in Nile tilapia. A El-Kader, F Marwa, AF Fath El-Bab, MF Abd-Elghany, A-WA Abdel-Warith, EM Younis and MAJBTER Dawood [89] found that the values of total serum protein and globulin were significantly higher in fish fed 0.25 and 0.5 mg nano-Se kg^− 1^.

SY has powerful antioxidant activity and participates in the antioxidant defense system and immune system moderation, and it acted directly as support for organismal health [23, 117]. Therefore, dietary Se prevents the toxic effects of malathion by ameliorating oxidative damage and enhancing physiological alterations that may affect fish health. Moreover, it improved normal feeding, food assimilation, metabolism, and growth in *Oreochromis niloticus*, minimizing the hazards associated with pesticide exposure [82]. Besides, R Alvarez, A Morales and A Sanz [118] reported that organic selenium had an advantage in reducing oxidative stress and is incorporated into kidneys, liver, and gastrointestinal mucosa proteins as selenomethionine and selenocysteine and is an essential micronutrient for fish. It was shown that several diseases are associated with increased expression of protein sulfhydryl groups (−SH), which were then oxidized to inactive disulfides bonds by selenium (S-S) [119].

Generally, Se played a major cell reinforcement component due to its incorporation in selenocysteine in enzyme glutathione peroxidase (GPx), GPx scavenges H_2_O_2_ and lipid hydroperoxides, using glutathione-reducing counterparts and protecting membrane lipids and macromolecules from oxidative damage and enhancing the body’s cell resistance and that acts against reactive oxygen species (ROS) [120, 121]**.** Because it is absorbed as an amino acid, selenomethionine is more easily assimilated into the body [51]. Similarly, A El-Kader, F Marwa, AF Fath El-Bab, MF Abd-Elghany, A-WA Abdel-Warith, EM Younis and MAJBTER Dawood [89], S Ghaniem, E Nassef, AI Zaineldin, A Bakr and S Hegazi [91] recorded a significant (*P* < 0.05) reduction in MDA levels in all selenium-supplemented fish groups compared with levels in the control.

## Conclusions

In conclusion, results of this study indicated that chronic exposure of *Oreochromis niloticus* to organophosphorus agrochemicals such as glyphosate (2 mg L^− 1^), malathion (0.5 mg L^− 1^) and their combination (1.6 mg L^− 1^ glyphosate and 0.3 mg L^− 1^malathion) resulted in detrimental effects on performance, hematobiochemical variables, as well as oxidative damage indicative parameters in liver and kidney tissues. Exposure to these agrochemicals’ residues may potentially harm the health of tilapia species as well as the health of human consumers, therefore, their use near a fish farm or in areas close to the aquatic environment should be discouraged. The addition of SY to the fish diet (3.3 mg kg^− 1^ diet organic selenium) ameliorated the fish performance and health status even in the presence of organophosphorus agrochemicals intoxication. Dietary inclusion of SY can be used as a sustainable bioremediation strategy that mitigates many of the negative effects of glyphosate/ malathion exposure in fish by ameliorating oxidative damage and enhancing the physiological alterations which may affect the health of fish. Future studies are needed to assess the toxic effects of these agrochemicals individually or in a mixture with dietary inclusion of organic selenium for other common freshwater and marine fish species cultured in Egypt.

## Data Availability

All data generated or analyzed during this study are included in this published article [and its supplementary information files].
